# Orthogeriatrics in Hip Fracture

**DOI:** 10.2174/1874325001711011181

**Published:** 2017-10-31

**Authors:** Helen Wilson

**Affiliations:** Royal Surrey County Hospital, Guildford, United Kingdom

**Keywords:** Hip Fracture, Orthogeriatric, Fragility Fracture, Peri-operative management

## Abstract

Orthogeriatrics is a system of care for complex elderly patients who have suffered fragility fractures. This has resulted in demonstrable improvements in care for patients with hip fracture. The article includes a review of the early history of orthogeriatrics as well as current concepts in orthogeriatric care including the use of national databases, audit and important aspects of clinical care such as analgesia, perioperative decision making, ceilings of care and rehabilitation.

## 1 INTRODUCTION

Orthogeriatrics or geriatric orthopaedics is the multi-disciplinary approach to the management of frail and often complex patients presenting with fragility fracture to ensure that they receive appropriate and timely intervention to allow the best possible outcome. An understanding of how the impact of trauma, anaesthesia and surgery will affect patients with physical and cognitive frailty is essential. This usually requires orthopaedic surgeons working along side geriatricians with an expertise in this area. It is important to recognise that more than a quarter of patients presenting with hip fracture are likely to be in their last year of life and priorities for these individuals require careful communication and consideration.

The last ten years have seen remarkable changes in the day to day management of patients with hip fracture in the UK and across the world. The passion of a few remarkable individuals with a belief that they could improve care for these often frail and complex patients has developed into a worldwide effort to support countries where the rapidly aging population is predicted to result in overwhelming increases in the numbers of patients presenting with hip fracture. Changing practice has been supported by an increasing evidence base and driven forward by a standardised national database in an increasing number of countries. This approach has been shown to improve outcomes, reducing mortality and improving secondary prevention [1].

The aims of this article are to review the history behind the development of Orthogeriatrics as well as the current systems and standards of care.

## 2 HISTORY OF ORTHOGERIATRICS IN THE UK

Patients with hip fracture were traditionally managed with up to three months of bed rest to allow the bone to heal and traction to restore leg length and to help with pain control. In the 1950s, particularly for younger patients, surgery became an option. Surgical intervention aims to improve pain, restore mobility and to reduce deformity. This has become the norm for the majority of patients in the UK, although sadly non-operative management remains common in developing countries.

Lionel Cosin is considered the founder of orthogeriatrics in the UK. He was described in his obituary as both a surgeon and geriatrician [2]. He trained as a general surgeon in the 1930s but his career progression was interrupted by the Second World War, when he found himself managing 300 beds of chronically sick patients who were not expected to improve. As new patients with hip fracture were admitted, deemed too old or frail for surgical treatment, Cosin decided to operate and offer active rehabilitation with good effect. He subsequently pursued a career in rehabilitation in Oxford and became one of the founding members of the British Geriatrics Society [3].

The concept and principles of Geriatric Orthopaedics were later described by Michael Devas, another Orthopaedic surgeon in the UK and published in the British Medical Journal in 1974 [4]. He worked in Hastings and regarded himself as a ‘humble carpenter’ but working alongside his colleague Bobby Irvine, a consultant geriatrician, he clearly came to understand the concepts of orthogeriatrics, which remain essentially unchanged years later.

The New York University Hospital for Joint Diseases in 1985 planned and initiated a similar geriatric hip fracture programme with an interdisciplinary research group comprising orthopaedic surgeons, geriatricians, physiotherapists, nursing staff, nutritionalists, social workers and epidemiologists. Their database helped to produce a number of research papers and their experience and philosophies were captured in Koval and Zucherman’s Hip Fracture: A practical Guide to management published in 2000 [5].

## 3 DATABASES AND AUDIT

The collection of data is essential for research, audit and quality improvement. The first hip fracture registry (RIKSHOFT) was set up by Karl-GoranThorngren from Lund University Hospital in Sweden in 1988. Following grants from the European Commission, the Standardised Audit of Hip Fractures (SAHFE) piloted data collection across European countries between 1995-1998.

The Scottish Hip Fracture Audit, based on RIKSHOFT, started in 1993 and collected data for 15 years resulting in a number of publications adding to the evidence base.

Despite growing evidence demonstrating improved outcomes, the routine involvement of geriatricians in the management of patients with hip fracture remained unusual. Patients were generally admitted under the care of orthopaedic teams and those with complex co-morbidity often developed complications resulting in ad hoc medical review with a lack of continuity of care.

Audit involves measurement of compliance with standards. In 2003 the first edition of ‘The Care of Patients with Fragility Fracture’, known as the Blue Book, was published in the UK [6]. This reviewed all the published evidence for the management of patients with hip fracture and for the first time outlined six standards of care (Table **1**).

These standards set an ambitious expectation of routine geriatric medicine review of all patients with hip fracture.

The revised edition was published in 2007, sponsored jointly by the British Orthopaedic Association and the British Geriatrics Society alongside the launch of the UK National Hip Fracture Database (NHFD). This database aimed to provide annual published data of compliance with standards of care allowing hospitals to review their own performance year on year and to benchmark against other hospitals [7].

The first report from this voluntary web-based data collection was published in 2009 [8]. It included data from 64 hospitals (just over a third of all eligible hospitals) on almost 13,000 patients (estimated to be about a fifth of all cases). Of those hospitals submitting data, only one in five had routine geriatric medical review of patients with hip fracture.

Hip fracture was recognised as a common, serious condition with significant morbidity and mortality. The NHFD quickly demonstrated variability in the standards of care across the country making it an ideal condition to pilot payment by results in the form of a Best Practice Tariff (BPT) [9].

Criteria were agreed broadly based on the blue book standards and included a requirement for a named orthogeriatrician to be involved in overseeing the hip fracture pathway and a senior geriatric review of patients with hip fracture within 72 hours of admission.

The NHFD was used to demonstrate which cases met the criteria for BPT and as such with significant financial reward to be claimed for patients meeting the criteria, there was a rapid improvement in data completeness and a significant increase in the number of Geriatricians involved in hip fracture management.

The most recent report published in September 2015 included data for 64000 hip fractures from all 180 eligible hospitals estimated to be 93% of all cases treated [10]. As such this database now offers a wealth of information about the management of patients with hip fracture in the UK.

**Fig. (1):**
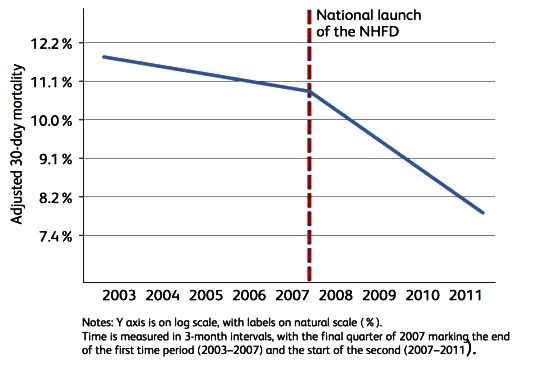
30 days mortality post hip fracture 2003-2011.

The latest facilities audit has demonstrated that most hospitals in England have a named orthogeriatrician routinely involved in hip fracture management but suggests considerable variation in staffing, process and structure [10]. Nevertheless, the NHFD has contributed to a positive impact on patient outcomes. (Fig. **1**)

## 4 ORGANISATION AND GOVERNANCE

The National Institute for Health and Clinical Excellence (NICE) guidelines on the management of patients with hip fracture were published in 2011 [11]. The advisory group reviewed all the evidence pertaining to the management of patients with hip fracture and published guidelines broadly in-line with the Blue Book standards but extending guidelines to reflect the whole pathway from admission through to discharge back into the community.

The guidelines recommend ‘a hip fracture programme’ including orthogeriatric assessment, rapid optimisation of fitness for surgery, and early identification of individual goals for multidisciplinary rehabilitation.

The Hip fracture programme should involve close collaboration between emergency services, orthopaedic surgeons, anaesthetists, orthogeriatricians, nursing and therapy teams with a named clinician from each of the above specialities responsible for developing and maintaining the hip fracture services.

The NHFD facilities audit from 2015 report suggests that 84% of hospitals report that they have regular scheduled clinical governance meetings with the majority having multidisciplinary representation to review and support continuous improvement [10].

It is important for a hip fracture programme to have written and agreed policies and protocols. A clear memorandum of understanding between teams ensures all are aware of their roles to prevent responsibilities falling between teams or indeed duplication. This is also useful in departments where junior doctors rotate frequently to aid induction.

As outlined by Irvine and Devas in their original model of orthopaedic geriatrics, it is essential that the ward environment is appropriate for managing frail older patients, many of who will have cognitive and sensory impairments. Health care professionals must be trained in interacting with such patients, understanding principles of geriatric nursing and actively engaging and involving family members for those who struggle to be independent.

The very nature of frail patients requires that many different specialists are involved in their care. This multidisciplinary approach works best in a hip fracture unit where all individuals learn from each other and develop an effective team through working together. It is essential to have a shared vision, positive attitude and a culture that embraces respect and shared responsibility. The team requires ward based leadership and co-ordination with a senior decision maker actively involved. The Ortho-geriatric Consultant is often best placed to be the senior decision maker supported by a specialist hip fracture nurse.

There has been increasing evidence to support clinical protocols with regard to pre-operative, intra-operative and post-operative management of patients with hip fracture [12]. These may vary in individual hospitals but should be agreed locally and be readily available. It is helpful to consider an orthogeriatric handbook which contains written details of standardised protocols or instructions on where to find them.

Teams often develop effectively through shared training, education, audit and quality improvement projects bringing members together away from the clinical environment to discuss, agree and work towards a shared vision. There are a number of organisations holding regular conferences which include up to date evidence for hip fracture management. A list of some is included after the references although it should be pointed out that this is not comprehensive.

With many patients having significant cognitive impairment, the multidisciplinary team has an advocacy role in supporting patients, families, carers and next of kin in ensuring appropriate decisions are made with regard to health and welfare. This requires a clear understanding of frailty, appropriate ceilings of care, the Mental Capacity Act and complex discharge planning.

## 5 THE CLINICAL PATHWAY

### 5.1 Assessing the Patient

Standardised recording of information in a clinical proforma may help multidisciplinary teams to find information easily and communicate effectively. This also helps to reduce duplication in terms of information gathering and supports data collection for audit purposes.

Frail patients benefit from Comprehensive Geriatric Assessment (CGA). This involves a holistic, multidimensional, interdisciplinary assessment of an individual by a number of specialists of many disciplines in older people’s health and has been demonstrated to be associated with improved outcomes in a variety of settings [13].

### 5.2 Pre-operative Assessment of Contributing Factors and Co-Morbidies

A significant proportion of patients presenting with hip fracture will have fallen as a result of a medical condition, for example; postural hypotension, cardiac syncope, intercurrent infection or acute stroke. A thorough history and examination is necessary to understand an individual’s current medical state. A baseline set of bloods and ECG should be routine with other investigations such as Chest xray, arterial blood gases or CT brain scan indicated in certain circumstances.

Patients with hip fracture require a rapid but thorough pre-operative assessment. There should be a clear understanding of medical co-morbidities and the impact that each has on the individual. For example, a diagnosis of Chronic Obstructive Pulmonary Disease (COPD) may reflect a mild condition requiring daily use of inhalers but resulting in little in the way of symptoms. At the other end of the spectrum, COPD may be severe, requiring long term oxygen therapy and resulting in a significant impact on exercise tolerance and activities of daily living. Review of previous records and investigations can help to gain a clear understanding of co-morbidities and the implications for anaesthesia, surgery and recovery to enable a risks / benefits discussion to take place. For example, an understanding an individual’s history of stroke, whether from carotid artery disease, haemorrhage or atrial fibrillation can help with peri-operative risk stratification and to decide upon medication including anti-platelets, anticoagulants and anti-hypertensives in the peri-operative period.

About a quarter of patients with hip fracture are admitted with an established diagnosis of dementia [14]. Many more will have a history suggestive of emerging cognitive impairment or undiagnosed dementia. Best practice tariff requires a pre-operative Abreviated Mini-mental Test Score (AMTS) to highlight cognitive impairment whether permanent or temporary. Patients with acute delirium or at risk of delirium need close monitoring in an appropriate environment to prevent prolonging symptoms and potentially poor outcome. Avoiding unnecessary bed moves, catheters and medication which can contribute to delirium is essential, along with daily review and monitoring of bladder, bowels, pain relief and nutrition as these individuals are unlikely to be able to identify or communicate all their needs.

### 5.3 Analgesia and Medication Review

Pain relief is the first aim of hip fracture management. Opiate analgesia is often required but should be used with caution due to side effects including respiratory depression, sedation, nausea, anorexia, urinary retention, constipation, dysequilibrium and confusion. Opiates are poorly tolerated in frail patients due to high incidence of side effects and reduced metabolism and excretion.

All healthcare professionals with prescribing and drug administering skills should be aware of risks of using opiates in frail patients. Communication between team members including paramedics, emergency department staff, theatre staff and ward teams is essential to ensure patients receive appropriate, effective analgesia without causing harm.

Intravenous paracetamol has been shown to be effective in reducing the need for opiates with minimal side effects [15]. It should be noted that in low weight patients the dose of paracetamol should be halved. Non-steroidal anti-inflammatories should be avoided as they may contribute to acute kidney injury and gastro-intestinal bleeding.

Over recent years there has been a move towards using early local nerve blocks for pain relief. Originally often used along with spinal anaesthesia, this simple technique has been trialled and shown to be effective in the emergency room reducing the need for opiate analgesia [16]. Although there is some debate about the effectiveness of Fascia-iliaca compartment block performed without ultrasound using the two pop technique, its use is increasing as it is a straight forward, cheap technique which is simple to learn. Front-line staff including paramedics and A&E staff may be trained to enable FICB to be used effectively in the emergency department [17]. It is important for hospitals to have a standardised technique and to audit effectiveness.

Medication review is one of the most important parts of the CGA. Polypharmacy is common in older patients and it is important to review all medications; firstly to understand the indication for each drug, to ensure that there is continued benefit but also to consider which medication to withhold in the immediate peri-operative period. Anti-hypertensives, hypo-glycaemics, anti-platelets and anticoagulants should be particularly reviewed as may cause problems around the time of surgery.

### 5.4 Decisions Around surgery and Its Timing

It is important to have access to early senior medical review for those with medical instability and to initiate treatment for reversible conditions early to prevent delays to surgery. The decision regarding the timing of surgery usually requires a senior orthogeriatrician, anaesthetist and orthopaedic surgeon to discuss and understand all aspects. It is important to consider whether the medical condition is likely to improve and the anticipated timescale for this, together with the type of operation required, likely blood loss and duration and type of anaesthetic. This must be weighed up against the likely effect on the patient of delaying surgery including complications of analgesia and immobility.

In most circumstances proceeding with early surgery is favoured. Non-surgical management is considered in a small number of patients. A trial of mobilisation may be appropriate in those with valgus impacted intracapsular fractures where the patient has little pain (although they must be warned that the fracture may become displaced requiring surgery at a later date). Surgery should be considered as a palliative approach in those reaching the end of their lives where prognosis is considered more than a few days to aid pain control and end of life nursing care.

### 5.5 Mortality and Ceilings of Care

The immediate peri-operative risk of mortality following anaesthesia and surgery is relatively small (less than one in a hundred) but thirty day mortality is about one in twelve [10]. The Nottingham Hip fracture score is a useful predictor of thirty day mortality taking into account age, cognition and co-morbidities [18]. Used together with the clinical picture, this is a helpful frailty score which can be reviewed when talking to patients and their relatives in setting realistic expectations for recovery.

Pre-operative discussions about appropriate ceilings of care are important. Frail older patients may not benefit from organ support or an intensive care environment; with poor physiological reserve they are unlikely to recover from significant organ failure. Do not attempt cardiopulmonary resuscitation decisions can be made pre-operatively, although many anaesthetists will rescind this decision during the anaesthetic as respiratory arrest may be drug induced and reversible under these circumstances [19].

## 6 POST-OPERATIVE CARE IN IMPROVING OUTCOMES

Ortho-geriatric management has supported early surgery for patients with hip fracture, recognising that reducing pain and restoring mobility as early as possible usually offers best possible outcome. Prioritising operating lists often now sees the frailest patient first on the list to ensure that they do not get cancelled due to theatre over-run. It has been recognised that hip fracture anaesthesia and surgery needs senior input to allow the safest and shortest anaesthetic and a quick and effective operation.

Post operative care should be aimed at minimising complications and allowing early mobilisation. NICE guidelines recommend that patients should be reviewed by physiotherapists on the day of or the day after surgery with a view to standing if possible [11]. From the NHFD it appears that three quarters of patients achieve this [10]. The number of patients achieving this target may be limited by resources, particularly at weekends and by patient factors including hypotension (often due to the inappropriate prescription of usual antihypertensive medication in the twenty-four hours before surgery), blood loss or side effects of opiates.

Frail patients are often malnourished and sarcopenic with fragile skin. Clear protocols to ensure adequate nutrition and pressure care from the point of admission need to be embedded. It is essential to have dieticians and experienced nurses involved in developing local protocols. Urinary catheters may be used on admission if toileting is painful and to protect the surgical field from contamination. Catheters should be removed as soon as possible post-surgery to reduce the risk of catheter associated infection and delirium.

Most patients become constipated in hospital as a result of relative immobility, dietary changes and opiate analgesia. Bowels should be monitored and pro-actively managed.

### 6.1 Rehabilitation and Discharge Planning

The average length of stay in an acute hospital bed for patients with hip fracture is about 18 days. This may vary from three to seven days in those fit and well with a good support network at home to months in those who require slow-stream rehabilitation. Resources in terms of community rehabilitation beds and home based rehabilitation teams vary significantly across the country. The NICE guidelines suggest that the hip fracture programme should remain involved with patient care until an individual has reached their baseline at which point they are discharged back to primary care [11].

Discharge planning is often complex as hip fracture may represent a frailty crisis from which an individual may not fully recover. Recognising when on-going medical intervention and active physiotherapy have reached their limits can be difficult and may require a period of time to achieve ‘medical stability’ and consistency. In some this will never be achieved and agreeing when discharge from a hospital environment is appropriate requires careful discussion with the patient / next of kin. Where possible these discussions should be recorded with plans for the future documented in an anticipatory care plan to ensure that an individual receives appropriate on-going care after discharge. This should be agreed with the patient, next of kin and primary care team to give guidance for the management of further medical instability or crisis.

A significant proportion of patients require an increase in long term care and the funding and sourcing of this takes time to be agreed and organised. Working closely with discharge co-ordinators and social services, predicting likely outcomes and timescales may help to set expectations for patients and families and reduce overall hospital length of stay.

### 6.2 Addressing Falls Risks and Cause of Fragility Fracture

The most significant risk factor for hip fracture is a previous fragility fracture. The opportunity to address falls risk and review bone health must not be missed. More than 200 randomised controlled trials exist in reviewing falls assessment and prevention but the challenge is in interpreting the literature and implementing effective strategies [20]. Whilst the falls assessment should be standardised, the approach to preventing further falls must be individualised. Falls assessment should include a detailed history of recent falls with any suggestion of syncope highlighted. As a minimum, patients should have a simple cardiovascular examination including lying and standing blood pressure measurement, a 12 lead ECG and auscultation of heart sounds to look for aortic stenosis. Medication review is an essential part of falls assessment. Medication often contributes to postural hypotension. Sedatives and psychotropic medication are strongly linked to falls and require review and slow withdrawal if possible. A brief assessment of vision should be routine. Identification of new visual impairment and cataracts should initiate referral to ophthalmology where appropriate. Bifocal and multifocal glasses can contribute to trips should be reviewed [21].

Gait and balance assessment with targeted exercise programmes may also reduce falls and fractures but provision of community services is variable and compliance is often poor [22]. Review of footwear and home environment may help to reduce falls and fractures in those at high risk [23].

A fragility fracture should also initiate a review of bone health. About 50% of those presenting with a hip fracture will have underlying osteoporosis. NICE guidelines recommends treating all those over 75 years who present with a hip fracture [24]. Secondary causes for low bone mass should be considered reviewing history of malabsorption or medication associated with osteoporosis such as steroids, anticonvulsants and LHRH analogues. Vitamin D deficiency should be corrected.

Those that are able to undergo a DEXA scan should do so both to establish a diagnosis but also to review response to treatment. First line treatment is with bisphosphonates. These can be administered orally on a daily, weekly or monthly basis or by intravenous infusion annually. Alternatives such as Denosumab or Teriperatide are usually reserved for those unable to take bisphosphonates or with complex disease.

Other causes of pathological fractures should be also considered – this includes metastatic solid organ malignancy, primary bone or haematological malignancies and other benign bone disorders e.g. Pagets disease.

### 7 OTHER FRAGILITY FRACTURES

While the focus of this article is on orthogeriatric management of hip fracture, many of the principles discussed can be applied to patients presenting with other fragility fractures, particularly those requiring surgical fixation. In the future orthogeriatrics may expand to play an important role in the care of this much larger group of patients. For those managed non-operatively on-going care may be on acute geriatric medicine wards, in rehabilitation settings or in the community. It is essential to have a system in place to ensure all have access to falls and bone health assessment usually overseen by a fracture liaison service. This may be based in primary or secondary care but should link in with falls clinics, orthogeriatricians and often rheumatologists or endocrinologists for younger patients. A feasibility report with regard to developing a database similar to the NHFD for other fragility fractures has recently been published [25]. Internationally standards have been set with self-accreditation of Fracture Liaison Services available through the Capture the Fracture campaign [26].

## CONCLUSION

Orthogeriatrics encompasses the multidisciplinary team approach to fragility fracture management. This involves the co-ordination of healthcare professionals including paramedics, the emergency room staff, theatres, anaesthetists, orthopaedic surgeons, geriatricians, nursing staff, physiotherapists, dieticians, occupational therapists, discharge co-ordinators, social services, rehabilitation teams and primary care teams. Standardisation of the hip fracture pathway with agreed protocols and guidelines improves overall care. However, it is essential to recognise each patient’s individual history and to try and understand their priorities, working with them and their next of kin to ensure their best possible outcome.

## Figures and Tables

**Table 1:** British orthopaedic association/british geriatrics society blue book standards for hip fracture care.

1. All patients with hip fracture should be admitted to an acute orthopaedic ward within 4 hours of presentation.
2. All patients with hip fracture who are medically fit should have surgery within 48 hours of admission, and during normal working hours.
3. All patients with hip fracture should be assessed and cared for with a view to minimising their risk of developing a pressure ulcer
4. All patients presenting with a fragility fracture should be managed on an orthopaedic ward with routine access to acute orthogeriatric medical support from the time of admission.
5. All patients presenting with fragility fracture should be assessed to determine their need for antiresorptive therapy to prevent future osteoporotic fractures.
6. All patients presenting with a fragility fracture following a fall should be offered multidisciplinary assessment and intervention to prevent future falls.

## LIST OF ABBREVIATIONS

AMTS: = Abbrieviated Mental Test Score
BPT: = Best Practice Tariff
CGA: = Comprehensive Geriatric Assessment
COPD: = Chronic Obstructive Pulmonary Disease
CT: = Computerised Tomography
DEXA: = Dual Energy Xray Absorbitometry
ECG: = Electrocardiogram
FICB: = Fascio-Iliaca Compartment Block
NHFD: = National Hip Fracture Database
NICE: = National Institute of Clinical Excellence
SAFHE: = Standardised Audit of Hip Fractures Europe
